# SP1-upregulated LBX2-AS1 promotes the progression of glioma by targeting the miR-491-5p/LIF axis

**DOI:** 10.7150/jca.63289

**Published:** 2021-10-11

**Authors:** Wentao Li, Ismatullah Soufiany, Xiao Lyu, Chenfei Lu, Yutian Wei, Zhumei Shi, Yongping You

**Affiliations:** 1 Department of Neurosurgery, The First Affiliated Hospital of Nanjing Medical University, Nanjing 210029, Jiangsu, China.; 2 Institute for Brain Tumors, Jiangsu Key Lab of Cancer Biomarkers, Prevention and Treatment, Jiangsu Collaborative Innovation Center for Cancer Personalized Medicine, Nanjing Medical University, Nanjing 211166, Jiangsu, China.

**Keywords:** LBX2-AS1, miR-491-5p, LIF, EMT, Glioma

## Abstract

**Background:** Mounting evidences have shown the importance of lncRNAs in carcinogenesis and cancer progression. LBX2-AS1 is identified as an oncogenic lncRNA that is abnormally expressed in gastric cancer and lung cancer samples. This study aims to explore the potential role of LBX2-AS1 in regulating proliferation and EMT in glioma, and the underlying mechanism.

**Methods:** Relative levels of LBX2-AS1 in glioma samples and cell lines were detected by qRT-PCR and FISH. *In vivo* and *in vitro* regulatory effects of LBX2-AS1 on proliferation and EMT were examined in the xenograft glioma model and glioma cells. The interaction between SP1 and LBX2-AS1 was assessed by ChIP. Through bioinformatic analyses, dual-luciferase reporter assay, RIP and Western blot, the regulation of LBX2-AS1 and miR-491-5p on the target gene LIF was identified.

**Results:** LBX2-AS1 was upregulated in glioma samples and cell lines, and its transcription was promoted by binding to the transcription factor SP1. As a lncRNA mainly distributed in the cytoplasm, LBX2-AS1 sponge miR-491-5p to further upregulate LIF. The subsequent activated LIF/STAT3 signaling was responsible for promoting proliferation and EMT in glioma.

**Conclusion:** LBX2-AS1 is upregulated by SP1 in glioma, which promotes the progression of glioma by targeting the miR-491-5p/LIF axis. In view of this, LBX2-AS1 is suggested as a novel diagnostic biomarker and therapeutic target of glioma.

## Introduction

Glioma characterized by local infiltration and neovascularization is the most common primary tumor of the central nervous system 1-3. Among which, glioblastoma (GBM) is the most malignant, accounting for 50% of gliomas, and the 5-year survival rate is less than 10% 4, 5. Glioma is known to be highly heterogeneous, even different cells from the same tumor can exhibit distinctive patterns of gene expression and biological behavior 6, 7. Therefore, it is a huge challenge to enhance the diagnostic accuracy of glioma, and thus performs targeted therapy to improve the clinical outcome.

LncRNAs (long non-coding RNAs) are over 200 nucleotides long, which are functional in epigenetic regulation, histone modification, transcription control and RNA metabolism 8, 9. Current evidences have proven the regulatory effects of lncRNAs on cancer progression 10, 11. L Salmena et al. first proposed the ceRNA theory, in which lncRNAs exert a sponge effect on miRNAs, therefore upregulating mRNAs downstream to them 12. Based on this theory, the lncRNA-miRNA-mRNA axis has been widely explored in multiple types of cancers. It is reported that MALAT1 upregulates VEGF, Slug and Twist by exerting the sponge effect on miR-126-5p, thus triggering EMT and angiogenesis in colorectal cancer 13. Serving as a vital ceRNA, CRNDE activates the PIWIL4/STAT3 signaling pathway through miR-384, which further accelerates the proliferative and invasive capacities of glioma cells 14.

Recently, LBX2-AS1 has been confirmed as an oncogenic lncRNA 15-17. LBX2-AS1 is also upregulated in glioma samples and correlated to patient prognosis, but its role in glioma remains unclear 18, 19. This study first detected the differential level of LBX2-AS1 in glioma tissues, its oncogenic role and the underlying mechanism were further explored.

## Materials and Methods

### Collection of GBM samples

All the clinical specimens were collected from patients in the Department of Neurosurgery, The First Affiliated Hospital of Nanjing Medical University during 2019-2020. Thirty samples of GBM were collected during surgery, including 20 primary cases and 10 recurrent cases. All the patients were confirmed by postoperative pathological diagnosis. The pathological diagnosis was based on the 2007 World Health Organization classification of central nervous system tumors. Moreover, none of the patients had a history of other tumors except glioma, which was confirmed by preoperative imaging examination. In addition, 5 normal brain specimens were collected during decompression surgery of traumatic brain injury. All the tissues were collected after participants signed written informed consent. This study was approved by the Clinical Research Ethic Committee of the First Affiliated Hospital of Nanjing Medical University (Ethics number: 2019-SR-479).

### Bioinformatic analyses

RNA-seq data were downloaded from The Cancer Genome Atlas (TCGA, https://portal.gdc.cancer.gov/), Chinese Glioma Genome Atlas (CGGA, www.cgga.org) and Gene Expression Omnibus (GEO, https://www.ncbi.nlm.nih.gov/geo) for bioinformatic analyses. GO and KEGG were performed using DAVID (https://david.ncifcrf.gov/). The limma R package and clusterprofiler package were used for differential analysis and GSEA, respectively.

### Cell culture

Human glioma cell lines (U87, LN229, A172, T98G, U251), normal human astrocytes (NHA) and human umbilical vein endothelial cells (HUVECs) were provided by the American Type Culture Collection (ATCC). Primary GBM cell line (N3) was gifted from Beijing Tiantan Hospital. HEK293T cell line was provided by Cell Bank of the Chinese Academy of Sciences. HUVECs were cultured in EGM-2 (Lonza), and the remaining were cultivated in DMEM containing 10% FBS. Cells were incubated at 37 °C in a humidified environment containing 5% CO_2_.

### Cell transfection

Transfection of siRNAs, miRNA mimics and plasmids (Genechem, Shanghai, China) was conducted using Lipofectamine 2000 (Invitrogen). cDNAs that were complementary paired to LBX2-AS1, SP1 and LIF were synthesized, which were cloned into pcDNA3.1 (Invitrogen). LBX2-AS1 shRNAs and negative control (sh-NC) were synthesized by Genechem (Shanghai, China). Screening by puromycin at 48 h, stably expressed N3 and U87 cell lines were established. Small interfering RNA (siRNA) and short hairpin RNA (shRNA) sequences designed for specific targets are listed in Table S1-3.

### qRT-PCR

Cells were lysed in TRIzol (Invitrogen, CA, USA) and the isolated RNAs were reversely transcribed to cDNAs using the PrimeScript RT (Takara, Nanjing, China). PARIS^TM^ Nuclear/Cytosol Fractionation Kit was used for isolating nuclear and cytoplasmic components. After preparing a PCR system using the SYBR Green Premix Ex Taq (Takara, Nanjing, China), PCR was conducted with U6 and GAPDH as the internal references of nuclei and cytoplasm, respectively. Relative level was calculated using 2^-ΔΔCt^ method. Primer sequences were shown in Table S4.

### Fluorescence *in situ* hybridization (FISH)

RNA-FISH was conducted as previously described 20. LBX2-AS1 probe was provided by RiboBio (Guangzhou, China). FISH-RNA signal in glioma cells and specimens were captured using a confocal microscope system (Zeiss LSM 700).

### Transwell assay

For invasion assays, Transwell inserts (Corning, New York, USA) were pre-coated with 20 μg/μl Matrigel (BD Biosciences, New Jersey, USA). 2×10^4^ cells suspended in serum-free medium and culture medium containing 10% FBS were respectively applied at the top and bottom of the prepared insert. After 24h, cells invaded from the top to the bottom were fixed in 4% paraformaldehyde for 10 min and dyed in 0.1% crystal violet for 30 min. Invasive cells in 3 random fields per sample were captured for counting (scale bar = 100 µm). Cell migration assay were similarly conducted in Transwell inserts without pre-coating Matrigel.

### Cell proliferation assay

Proliferative potential of glioma cells was assessed by CCK-8 and colony formation. In the first experiment, cells were seeded in a 96-well plate and absorbance at 450 nm was measured using Cell Counting Kit-8 (Dojindo, Shanghai, China). In colony formation assay, cells were seeded in a 6-well plate and cultivated for 14 days. Colonies were washed in PBS twice, fixed in 4% paraformaldehyde for 10 min and stained in 0.1% crystal violet for 30 min, which were captured under a microscope.

### Dual-luciferase reporter assay

Promoter-containing vector for LBX2-AS1, SP1, TEAD2 and KLF5 (Genechem, Shanghai, China) were co-transfected into HEK293T cells, respectively. P1-wt (wild-type) and P1-mut (mutant) sequences were synthesized and cloned into pGL3-basic luciferase vectors (Promega, Madison, USA), which were co-transfected into HEK293T cells with SP1 plasmid. In addition, luciferase vectors containing mutant or wild-type sequences in which miR-491-5p bound to LBX2-AS1 or LIF were co-transfected into N3 or U87 cells with either miR-491-5p mimic or negative control. Relative luciferase activity was measured using the Promega Dual-luciferase Reporter System, and normalized to that of Renilla luciferase activity.

### Chromatin immunoprecipitation (ChIP)

EZ-ChIP Kit (Millipore, Billerica, MA, USA) was used for ChIP assay. Briefly, cells were cross-linked in 1% formaldehyde for 10 min and terminated by glycine. Cells were lysed to chromatin fragments by sonication. DNA-protein complex was incubated with 3 μg anti-SP1 (Cell Signaling Technology, 9389). Anti-IgG (Millipore, 12-371) was used as the negative control. The special primers for P1 site, P2 site and P3 site were listed in Table S5.

### RNA immunoprecipitation (RIP)

Magna RIPTM RNA-Binding Protein Immunoprecipitation Kit (Millipore, Billerica, MA, USA) was used for RIP assay. Briefly, cell lysate was incubated with anti-Ago2 (Abcam, ab32381), and anti-IgG (negatively control). A protein-RNA complex was captured, followed by removal of the protein. The magnetic beads were repeatedly washed with RIP washing buffer, and the immunoprecipitated RNA was quantified by performing qRT-PCR.

### Western blot

Western blot was conducted as previously described 21. The primary antibodies are listed in Table S6.

### Immunohistochemistry (IHC)

After sacrifice, tumor tissues collected from nude mice were fixed in 4% paraformaldehyde, and paraffin embedded for preparation of tissue sections. Sections prepared from *in situ* tumors were incubated with anti-Ki-67, anti-LIF and anti-p-STAT3, and those prepared from subcutaneous tumors were incubated with anti-Ki-67, anti-N-cadherin, anti-E-cadherin and anti-Vimentin. IHC images were finally captured under a microscope (scale bar = 50 µm). The quantification of IHC staining was performed according to the proportion of positively stained tumor cells and the intensity of staining. The scores for proportion of positively stained tumor cells (0 = 0%, 1 = 0.01-25%, 2 = 25.01-50%, 3 = 50.01-75%, 4 =75.01-100%) and intensity (0 = negative, 1 = weak, 2 = moderate, and 3 = strong staining) were added to obtain IHC overall staining score. IHC overall staining score = staining intensity × proportion of positively stained tumor cells.

### Immunofluorescence assay

Cells were seeded into 24-well plates, fixed in 4% paraformaldehyde, and permeabilized with 0.1% Triton X-100, followed by blocking with 1% BSA. Then, cells incubated with primary antibodies at 4 °C overnight, including anti-GFAP (Cell Signaling Technology, 3670), anti-p-STAT3 (Abcam, ab76315). Subsequently, cells were incubated with appropriate secondary antibody (Alexa Fluor® 594, ab150080; Alexa Fluor® 488, ab150113) for 1 h and the nuclei stained by DAPI. Images were captured under fluorescence microscope (scale bar = 50 µm).

### *In vivo* assay

6-week-old male BALB/c nude mice were provided by Animal Core Facility of Nanjing Medical University. Twelve mice were used in the subcutaneous xenograft model, with 6 mice in each group. They were respectively subcutaneously implanted with 1×10^7^ U87 cells transfected with sh-NC or sh-LBX2-AS1#1. In the orthotopic xenograft model, 12 mice were randomly assigned to two groups, with 6 in each. 2.5×10^5^ luciferase-labeled U87 cells transfected with sh-LBX2-AS1#1 or sh-NC were stereotactically implanted in nude mice using a stereotaxis instrument. Bioluminescence imaging of orthotopic tumors in mice was examined using optical imaging system (IVIS spectrum, PerkinElmer, USA).

### Statistical analysis

Statistical analyses were conducted using GraphPad software version 8.0 (GraphPad software, San Diego, CA, USA) or SPSS Statistics 23.0 (SPSS, Chicago, IL, USA). Differences between groups were compared by the Student's *t* test or one-way ANOVA. Pearson's correlation test was performed for evaluating the correlation between two indexes. Kaplan-Meier survival analysis was performed, and the difference was compared by log-rank test. All experiments were repeated in triplicate, and data were expressed as mean ± standard error of the mean (SEM). A significant difference was considered at *p*<0.05 (*p<0.05, **p<0.01, ***p<0.001).

## Results

### Upregulation of LBX2-AS1 in glioma databases

**Figure 1:**
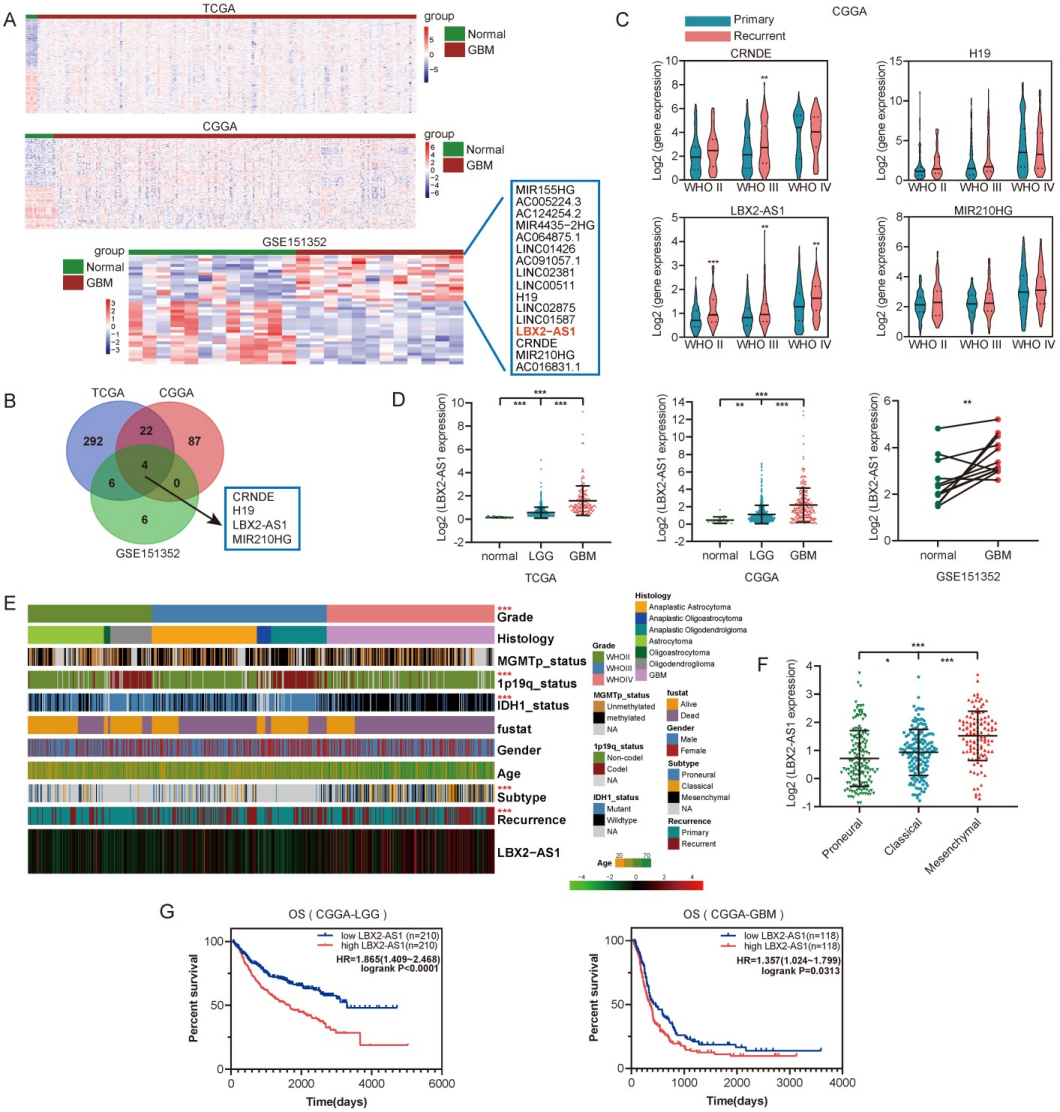
** Upregulation of LBX2-AS1 in glioma databases. (A)** Hierarchical cluster analysis of differential lncRNAs between normal and GBM samples in TCGA, CGGA and GSE151352 datasets. **(B)** Insertions of differentially expressed lncRNAs from three databases. **(C)** Relative levels of LBX2-AS1, CRNDE, H19 and MIR210HG in primary and recurrent glioma samples. **(D)** Relative levels of LBX2-AS1 in glioma samples from TCGA, CGGA and GSE151352 datasets. **(E)** Heatmap of the associations between LBX2-AS1 and clinicopathological features of glioma in CGGA dataset. **(F)** Relative levels of LBX2-AS1 in glioma samples from CGGA dataset categorized by transcription subtypes. **(G)** Kaplan-Meier survival analysis of LGG and GBM patients based on LBX2-AS1 in CGGA dataset. *p<0.05, **p<0.01, ***p<0.001.

To identify differentially expressed lncRNAs in glioma, we analyzed RNA-seq data from TCGA, CGGA and GEO (GSE151352) databases using the limma R package (FDR < 0.05, |Log_2_FC| >1). Differentially expressed lncRNAs in GBM samples were illustrated in the heatmaps and volcano plots (Figure 1A, Figure S1A). After taking the intersection, LBX2-AS1, CRNDE, H19 and MIR210HG were predicted upregulated in all the three databases (Figure 1B). We subsequently analyzed their expression levels in 693 glioma samples from CGGA database, and LBX2-AS1 was the only one that was differentially expressed between primary and recurrent cases of both low-grade glioma (WHOII, WHOIII) and GBM (Figure 1C). In addition, LBX2-AS1 level was higher in GBM than that in LGG (Figure 1D). By assessing clinical features of glioma samples in TCGA and CGGA databases, LBX2-AS1 was highly expressed in mesenchymal (MES) subtype and recurrent cases (Figure 1E, F, Figure S1B-D). Kaplan-Meier survival analysis obtained the conclusion that LBX2-AS1 was unfavorable to the overall survival of LGG and GBM patients (Figure 1G). Collectively, LBX2-AS1 was upregulated in glioma, which was correlated to the poor prognosis of glioma patients.

### Upregulation of LBX2-AS1 in glioma cell lines and specimens

**Figure 2:**
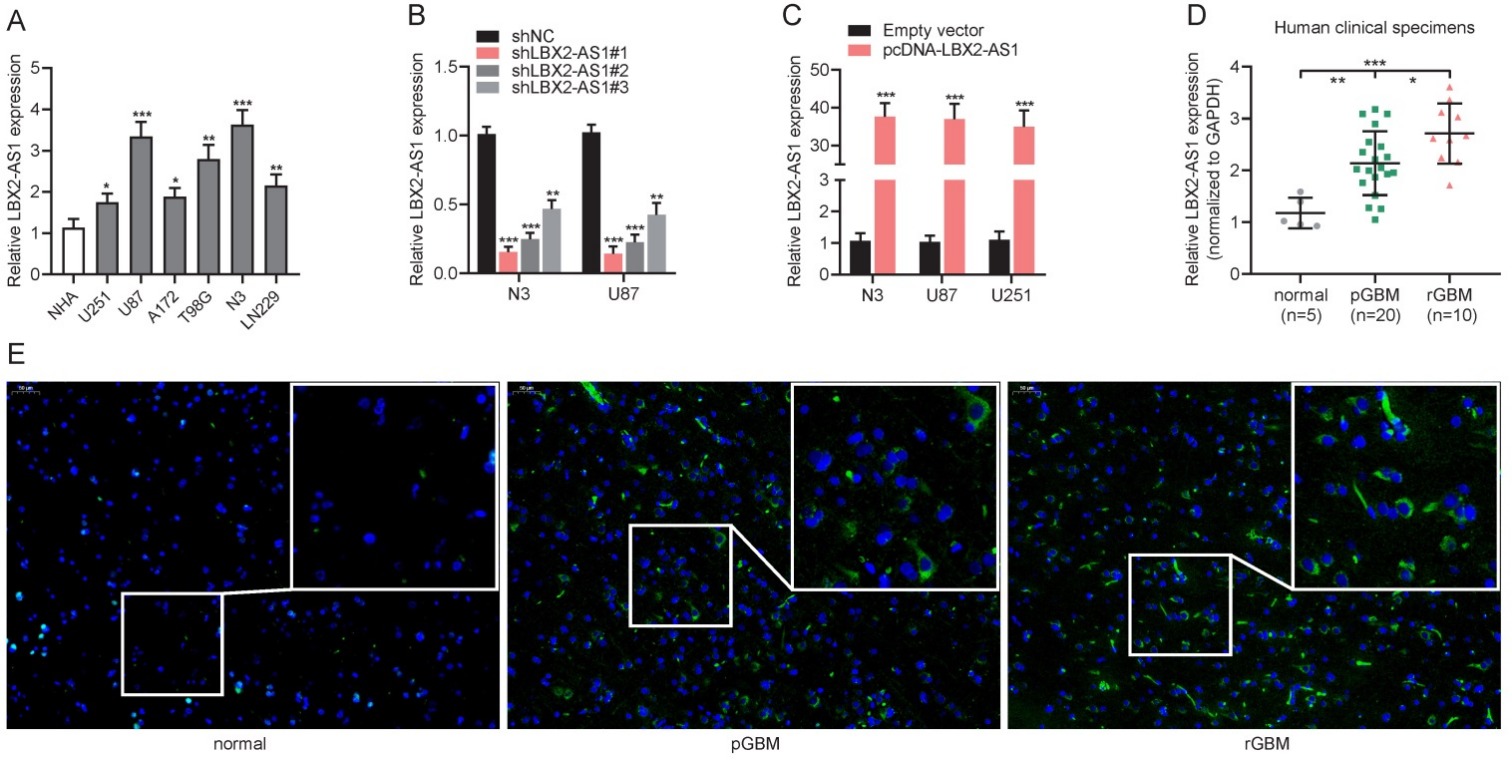
** Upregulation of LBX2-AS1 in glioma cell lines and specimens. (A)** Relative levels of LBX2-AS1 in normal human astrocyte cell line (NHA) and glioma cell lines (U251, U87, A172, T98G, LN229 and N3) detected by qRT-PCR. **(B)** Transfection efficacy of sh-LBX2-AS1#1, sh-LBX2-AS1#2 and sh-LBX2-AS1#3 in N3 and U87 cells detected by qRT-PCR. **(C)** Transfection efficacy of pcDNA-LBX2-AS1 in N3, U87 and U251 cells detected by qRT-PCR. **(D)** Relative levels of LBX2-AS1 in normal brain, primary and recurrent GBM specimens. **(E)** Positive expressions of LBX2-AS1 in normal brain, primary and recurrent GBM specimens measured by RNA-FISH. Scale bar = 50 μm. *p<0.05, **p<0.01, ***p<0.001.

Relative levels of LBX2-AS1 in normal human astrocytes (NHA), glioma cell lines (U251, U87, A172, T98G and LN229) and primary GBM cell line (N3) were measured by qRT-PCR (Figure 2A). In the following experiments, LBX2-AS1 highly expressed cell lines N3 and U87, and LBX2-AS1 lowly expressed cell line U251 were selected as *in vitro* objects. Then, we synthesized three LBX2-AS1 shRNAs and tested their transfection efficacy in N3 and U87 cells. At last, sh-LBX2-AS1#1 was selected because of its excellent performance (Figure 2B). Moreover, transfection of pcDNA-LBX2-AS1 markedly upregulated LBX2-AS1 in N3, U87 and U251 cells (Figure 2C). As expected, LBX2-AS1 level was higher in GBM specimens collected in our center than that of normal brain specimens, and notably, it was remarkably higher in recurrent GBM compared with that of primary ones (Figure 2D). RNA-FISH for quantification of LBX2-AS1 in GBM samples consistently supported PCR results (Figure 2E).

### Knockdown of LBX2-AS1 suppresses proliferation and EMT in glioma *in vivo* and *in vitro*

**Figure 3:**
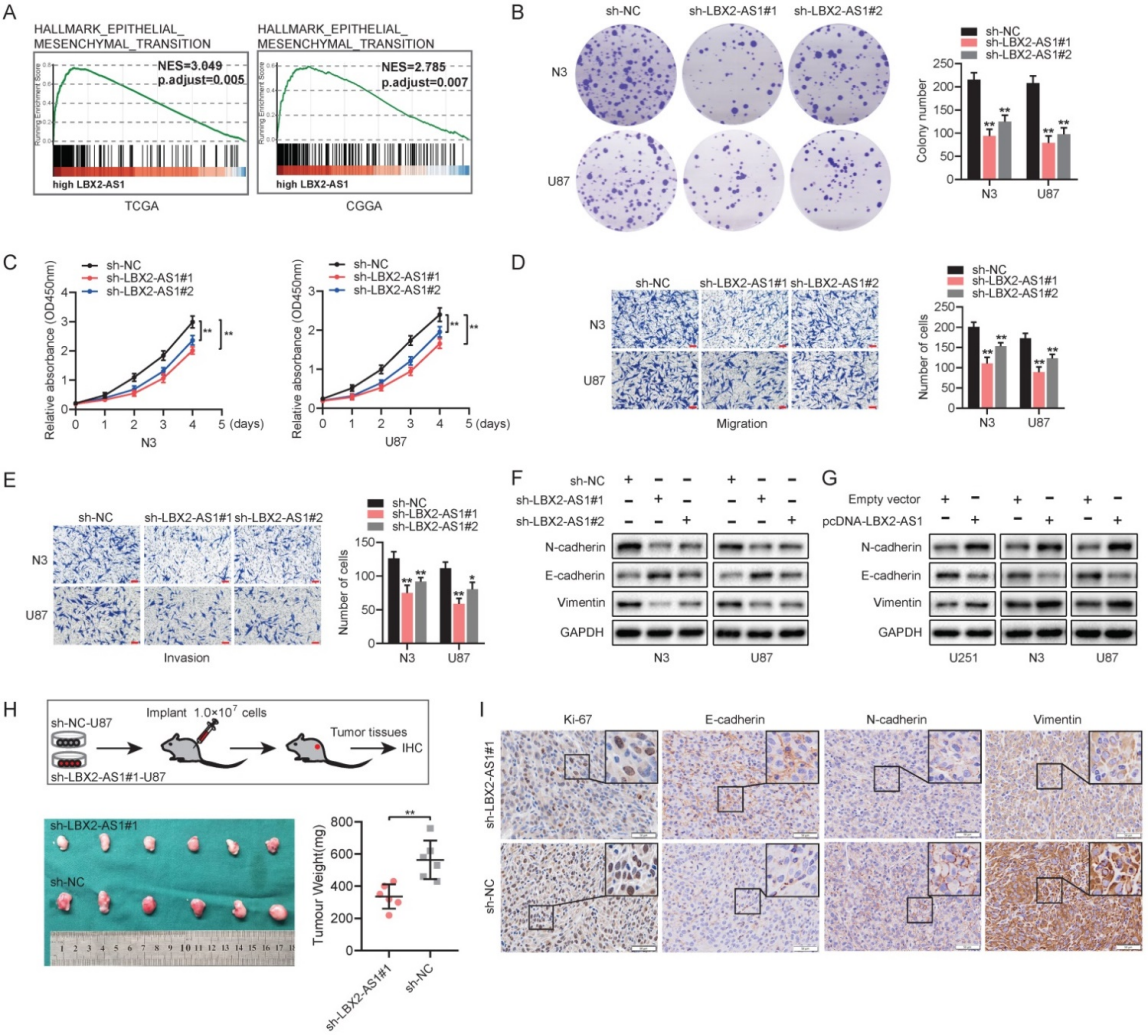
** Knockdown of LBX2-AS1 suppresses proliferation and EMT in glioma *in vivo* and *in vitro*. (A)** GSEA revealed the correlation between LBX2-AS1 and EMT in glioma. **(B, C)** Proliferation of N3 and U87 cells with LBX2-AS1 knockdown examined by colony formation assay and CCK-8 assay. **(D, E)** Migration and invasion of N3 and U87 cells transfected with sh-NC, sh-LBX2-AS1#1 or sh-LBX2-AS1#2. Scale bar = 100 μm. **(F, G)** Protein levels of EMT markers (N-cadherin, E-cadherin and Vimentin) in N3, U87 and U251 cells with knockdown or overexpression of LBX2-AS1. **(H)** Representative image of subcutaneously xenografted tumors in nude mice and scatter plots of tumor weight. **(I)** IHC staining of Ki-67, N-cadherin, E-cadherin and Vimentin in tumor sections. Scale bar = 50 μm. *p<0.05, **p<0.01.

According to the results yielded from GSEA, high-level LBX2-AS1 was correlated to epithelial-mesenchymal transition (EMT) in glioma (Figure 3A). Colony formation assay revealed that knockdown of LBX2-AS1 in N3 and U87 cells markedly reduced colony numbers, indicating the suppressed proliferative potential (Figure 3B). Cell viability of glioma cells was consistently reduced by transfection of sh-LBX2-AS1 (Figure 3C). Transwell assay obtained the conclusion that knockdown of LBX2-AS1 significantly inhibited migration and invasion of N3 and U87 cells (Figure 3D, E). We further detected protein levels of EMT markers in glioma cells regulated by LBX2-AS1. Western blot analyses showed that protein levels of N-cadherin, Vimentin were downregulated, while E-cadherin was upregulated in N3 and U87 cells with LBX2-AS1 knockdown (Figure 3F). Overexpression of LBX2-AS1 obtained opposite expression changes of them (Figure 3G). To assess the* in vivo* regulatory effects of LBX2-AS1 on tumorigenesis of glioma cells, we established a subcutaneous xenograft model in nude mice by implanting with U87 cells transfected with sh-NC or sh-LBX2-AS1#1, respectively. Xenografted tumor tissues were significantly lower in mice intervened with LBX2-AS1 knockdown than those of controls (Figure 3H). IHC images showed positive expression of E-cadherin in tumor sections was significantly higher in mice with LBX2-AS1 knockdown, and those of Ki-67, N-cadherin and Vimentin were lower than controls (Figure 3I). Taken together, knockdown of LBX2-AS1 suppressed cell proliferation and EMT in glioma.

### SP1 upregulates LBX2-AS1 in glioma

**Figure 4:**
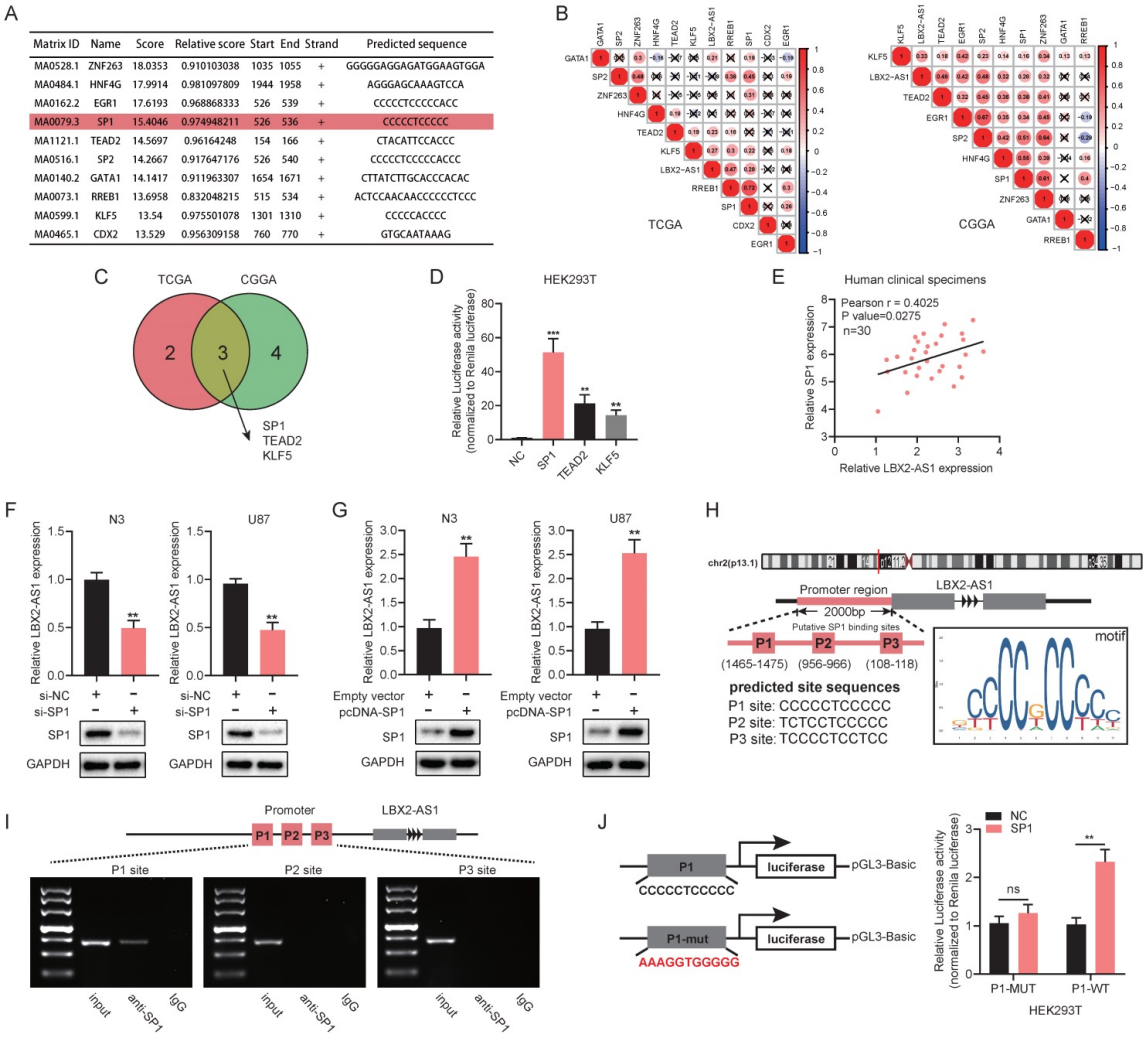
** SP1 upregulates LBX2-AS1 in glioma. (A)** The top 10 scored transcription factors that could bind to the promoter region of LBX2-AS1 were screened out using JASPAR. **(B)** Correlation between the expression levels of LBX2-AS1 and selected transcription factors in TCGA and CGGA databases. **(C)** Venn diagram showing three transcription factors (SP1, TEAD2, KLF5) that were intersected in both TCGA and CGGA databases by Pearson's correlation analysis (Pearson's r > 0.2). **(D)** Luciferase activity of the binding between three transcription factors and the promoter of LBX2-AS1. **(E)** Correlation between relative levels of LBX2-AS1 and SP1 in 30 clinical specimens of GBM. **(F, G)** Relative level of LBX2-AS1 in N3 and U87 cells with knockdown or overexpression of SP1. **(H)** Predicted sites where SP1 bound to the promoter of LBX2-AS1. The numbers below P1, P2 and P3 indicated the distance between these three binding sites and the transcription starting point of LBX2-AS1. **(I)** ChIP-PCR showing the enrichment of SP1 in P1 site of the promoter region of LBX2-AS1. **(J)** Dual luciferase reporter assay showing no significant influence of SP1 on the luciferase activity of P1-mut. **p<0.01, ***p<0.001.

We next explored the potential mechanisms underlying the abnormal expression of LBX2-AS1 in glioma. The top 10 scored transcription factors (TFs) that could bind to the promoter region of LBX2-AS1 were screened out using JASPAR (http://jaspar.genereg.net/) (Figure 4A). Then, we examined expression levels of the 10 selected TFs in TCGA and CGGA databases, and the correlation between expression levels of the selected TFs and LBX2-AS1 (Figure S2A, Figure 4B). At last, three TFs (Pearson's r > 0.2) were selected for further examinations, including SP1, TEAD2 and KLF5 (Figure 4C). Promoter-containing vector for LBX2-AS1 and TFs (SP1, TEAD2 and KLF5) were respectively co-transfected into HEK293T cells with negative control, followed by measurement of relative luciferase activity, and that of SP1 remained the highest (Figure 4D). Meanwhile, a positive correlation was detected between relative levels of SP1 and LBX2-AS1 in 30 clinical specimens, which was consistent with the findings obtained in TCGA and CGGA (Figure 4E, Figure S2B). Knockdown of SP1 markedly downregulated LBX2-AS1, while overexpression of SP1 upregulated it in N3 and U87 cells (Figure 4F, G, Figure S2C). Through analyzing ChIP-seq data of SP1 obtained from ENCODE database and putative binding sites of SP1 obtained from JASPAR, several predicted binding sites of SP1 were identified in the promoter region of LBX2-AS1 (Figure S2D). The top three scored potential sites, where SP1 bound to LBX2-AS1 were named as P1, P2 and P3 (Figure 4H). Later, ChIP assay confirmed that SP1 was enriched in P1 site in HEK293T cells (Figure 4I). After mutating P1 site, overexpression of SP1 did not significantly affect relative luciferase activity, proving the direct interaction between SP1 and LBX2-AS1 at P1 site (Figure 4J). Collectively, we considered that SP1 was responsible for the upregulation of LBX2-AS1 in glioma.

### LBX2-AS1 exerts a sponge effect on miR-491-5p

**Figure 5:**
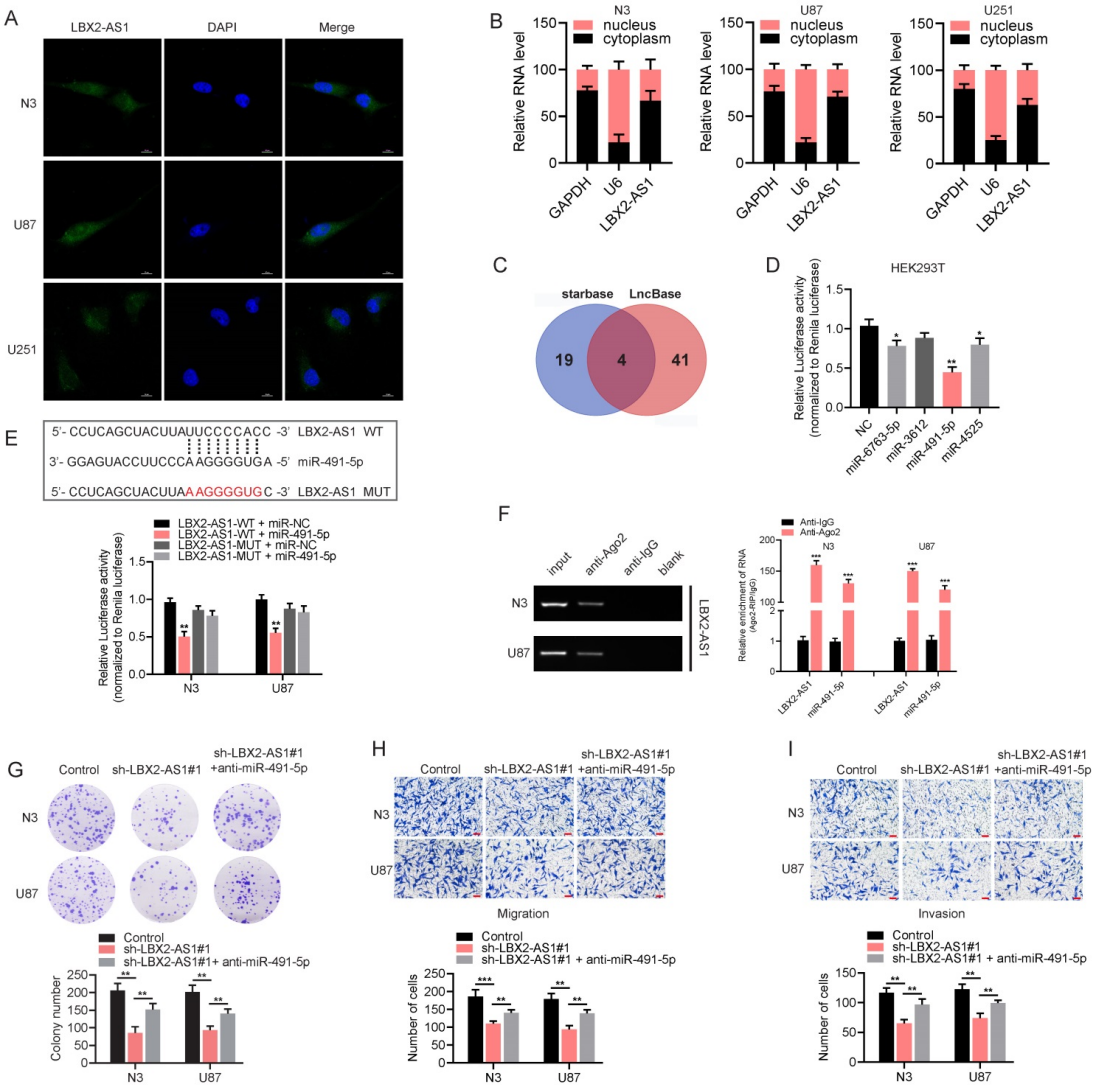
** LBX2-AS1 exerts a sponge effect on miR-491-5p. (A)** Localization of LBX2-AS1 in N3, U87 and U251 cells was examined by RNA-FISH. Scale bar = 10 μm. **(B)** Cytoplasmic and nuclear RNA levels of LBX2-AS1 in N3, U87 and U251 cells detected by qRT-PCR. GAPDH and U6 were the internal references for cell cytoplasm and nucleus, respectively. **(C)** Candidate miRNAs with complementary sites to LBX2-AS1 were predicted using starBase and LncBase, and 4 candidates were obtained from the intersection of the Venn diagram. **(D)** Luciferase activity of the pGLR-basic vector containing the sequence of LBX2-AS1 in HEK293T cells influenced by the 4 candidate miRNAs. **(E)** Luciferase activity of the pGLR-basic vector containing the sequence of LBX2-AS1 in N3 and U87 cells co-transfected with miR-491-5p mimics or negative control. **(F)** Co-immunoprecipitants of LBX2-AS1 and miR-491-5p in anti-Ago2 and anti-IgG. **(G)** Colony formation in N3 and U87 cells transfected with sh-LBX2-AS1#1 or miR-491-5p inhibitor + sh-LBX2-AS1#1. **(H, I)** Migration and invasion of N3 and U87 cells co-regulated by LBX2-AS1 and miR-491-5p. Scale bar = 100 μm. *p<0.05, **p<0.01, ***p<0.001.

In addition to epigenetic regulation in cell nuclei, lncRNAs distributed in cytoplasm can serve as endogenous competitors of certain miRNAs to regulate their target genes. RNA-FISH and subcellular fractionation PCR clarified that LBX2-AS1 was mainly distributed in the cytoplasm, suggesting the potential function of LBX2-AS1 as a ceRNA (Figure 5A, B). Then, we identified 4 candidate miRNAs with complementary sites to LBX2-AS1 using starBase (http://starbase.sysu.edu.cn/) and LncBase (http://carolina.imis.athena-innovation.gr/diana_tools/web/index.php) (Figure 5C). The predicted results were further validated by dual-luciferase reporter assay. Compared with control group, luciferase activity of vector containing the sequence of LBX2-AS1 was markedly reduced by overexpression of miR-491-5p in HEK293T cells (Figure 5D). After mutation of the complementary sites of miR-491-5p to LBX2-AS1, relative luciferase activity was unable to be affected by miR-491-5p, confirming the specific interaction between LBX2-AS1 and miR-491-5p in glioma cells (Figure 5E). Subsequently, RIP assay revealed that LBX2-AS1 and miR-491-5p could directly interact with Ago2 (Figure 5F), which is the core component of the RNA-induced silencing complex (RISC) 22. Moreover, the results of qPCR showed that miR-491-5p significantly affected the level of LBX2-AS1 in N3 and U87 cells (Figure S3). Finally, colony formation and Transwell assay showed that the suppressed proliferative, migratory and invasive potentials of N3 and U87 cells with LBX2-AS1 knockdown were partially reversed by silence of miR-491-5p (Figure 5G-I). It is concluded that LBX2-AS1 regulated cell proliferation and EMT in glioma through the sponge effect on miR-491-5p.

### LIF is the target mRNA of miR-491-5p and indirectly regulated by LBX2-AS1

**Figure 6:**
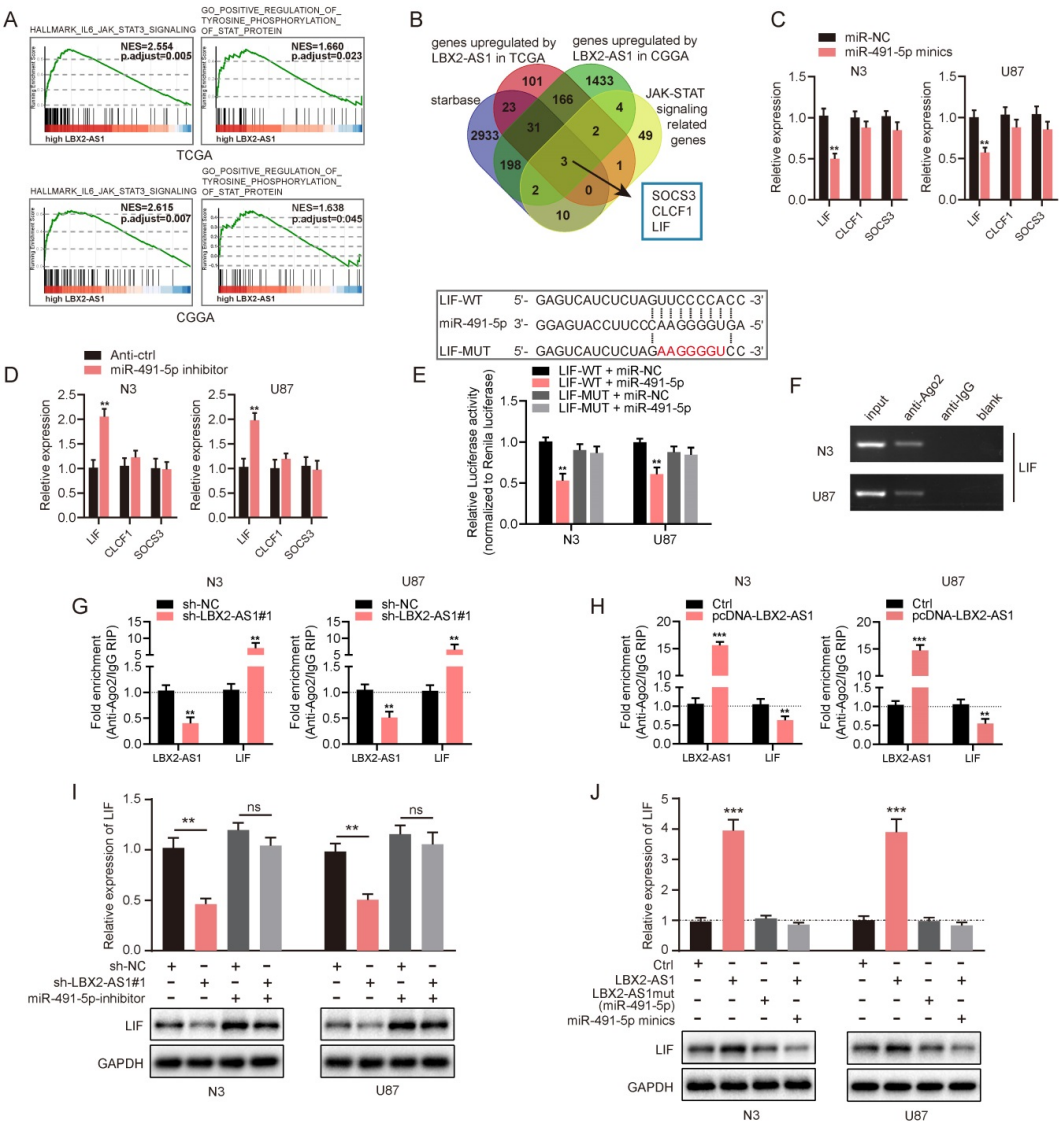
** LIF is the target gene of miR-491-5p and indirectly regulated by LBX2-AS1. (A)** GSEA revealed the correlation between LBX2-AS1 and the JAK-STAT3 signaling pathway. **(B)** Venn diagrams showing the selection of the target genes of miR-491-5p. **(C)** Relative levels of the three candidates in N3 and U87 cells transfected with miR-NC or miR-491-5p mimics. **(D)** Relative levels of three candidate targets of miR-491-5p in N3 and U87 cells with miR-491-5p knockdown. **(E)** Luciferase activity of LIF-WT and LIF-MUT in N3 and U87 cells transfected with miR-NC or miR-491-5p mimics. **(F)** Enrichment of LIF in anti-Ago2 and anti-IgG. **(G)** Enrichments of LBX2-AS1 and LIF in anti-Ago2 in N3 and U87 cells with LBX2-AS1 knockdown. **(H)** Enrichments of LBX2-AS1 and LIF in anti-Ago2 in N3 and U87 cells overexpressing LBX2-AS1. **(I, J)** Protein and mRNA levels of LIF in N3 and U87 cells co-regulated by LBX2-AS1 and miR-491-5p. **p<0.01, ***p<0.001.

The abovementioned results have confirmed the ceRNA function of LBX2-AS1, we thereafter identified the target gene of the LBX2-AS1/miR-491-5p axis in the ceRNA network. Through GSEA, GO and KEGG analyses, LBX2-AS1 was identified enriched in the regulation of the JAK-STAT3 signaling pathway (Figure 6A, Figure S4D). A total of 3 candidate genes were finally obtained after a comprehensive analysis on putative targets of miR-491-5p in starBase, upregulated genes regulated by LBX2-AS1 in TCGA and CGGA databases, and JAK-STAT3 signaling-related genes, including SOCS3, CLCF1 and LIF (Figure S4A-C, Figure 6B). qRT-PCR results showed that overexpression of miR-491-5p significantly downregulated LIF in N3 and U87 cells, and knockdown of miR-491-5p yielded the opposite results (Figure 6C, D). However, expression levels of SOCS3 and CLCF1 were not influenced by the regulation of miR-491-5p. Meanwhile, relative level of LIF was positively correlated to that of LBX2-AS1 in 30 clinical specimens, which were consistent with the correlation analyzed in TCGA and CGGA databases (Figure S4E-G). Dual luciferase reporter assay revealed that transfection of miR-491-5p mimics reduced luciferase activity of LIF-WT vector, whilst it did not influence that of LIF-MUT vector, indicating the direct binding of miR-491-5p in LIF 3'-UTR (Figure 6E). Furthermore, RIP assay confirmed the enrichment of LIF in anti-Ago2, and the recruitment of Ago2 in the LIF transcript was interestingly, negatively regulated by LBX2-AS1 (Figure 6F-H). It is suggested that LBX2-AS1 competed with the LIF transcript for Ago2-based RISC. We further assessed the regulatory effect of the LBX2-AS1/miR-491-5p axis on expression level of LIF by Western blot and qRT-PCR. Knockdown of LBX2-AS1 downregulated mRNA and protein levels of LIF in N3 and U87 cells, which was reversed by silence of miR-491-5p to a certain extent (Figure 6I). Overexpression of LBX2-AS1, conversely, upregulated mRNA and protein levels of LIF, which was abolished by overexpression of miR-491-5p. Notably, overexpression of LBX2-AS1 with mutant bindings sites to miR-491-5p did not influence the expression level of LIF (Figure 6J). Taken together, serving as a miR-491-5p sponge, LBX2-AS1 upregulated LIF in glioma cells.

### LBX2-AS1 advances malignant phenotypes of glioma *via* the LIF-STAT3 axis

**Figure 7:**
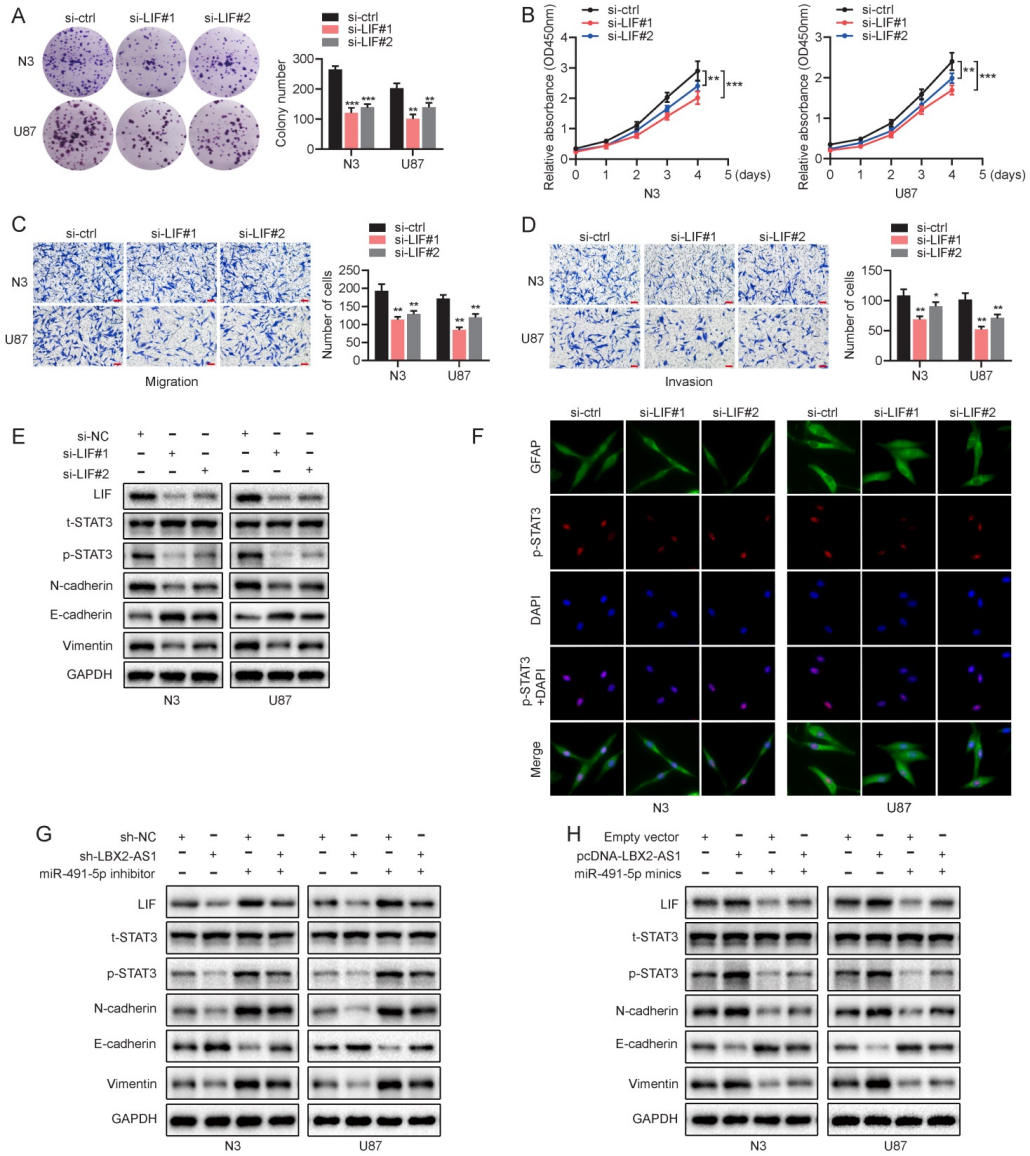
** LBX2-AS1 advances malignant phenotypes of glioma cells via the LIF-STAT3 axis. (A, B)** Proliferation of N3 and U87 cells transfected with si-NC or si-LIF detected by colony formation and CCK-8 assay. **(C, D)** Migration and invasion of N3 and U87 cells transfected with si-NC or si-LIF were examined by Transwell assay. Scale bar = 100 μm.** (E)** Protein levels of t-STAT3, p-STAT3 and EMT markers in N3 and U87 cells transfected with si-NC or si-LIF. **(F)** Immunofluorescence staining of p-STAT3 (red) in N3 and U87 cells transfected with si-NC or si-LIF. Scale bar = 50 μm. **(G, H)** Protein levels of LIF, t-STAT3, p-STAT3 and EMT markers in N3 and U87 cells regulated by miR-491-5p and LBX2-AS1. **p<0.01, ***p<0.001

We have confirmed a ceRNA network involving the LBX2-AS1/miR-491-5p/LIF axis in regulating proliferation and EMT of glioma. Its underlying mechanisms were then mainly investigated. Knockdown of LIF significantly attenuated proliferative, migratory and invasive abilities of N3 and U87 cells (Figure 7A-D). What's more, protein level of p-STAT3(Y-705) was remarkably downregulated in N3 and U87 cells with knockdown of LIF, which was consistently validated by immunofluorescence staining of p-STAT3(Y-705) (Figure 7E, F). It is concluded that knockdown of LIF reduced the level of p-STAT3 in nuclear. Moreover, LBX2-AS1 depletoin not only downregulated LIF and p-STAT3 in glioma cells, but also affected the protein levels of N-cadherin, E-cadherin and Vimentin (Figure 7G). Overexpression of LBX2-A1 yielded the opposite results (Figure 7H). Thus, we demonstrated that LBX2-AS1 promoted proliferation and EMT through LIF-STAT3 axis in glioma.

### Knockdown of LBX2-AS1 alleviates the growth of orthotopic glioma in nude mice

**Figure 8:**
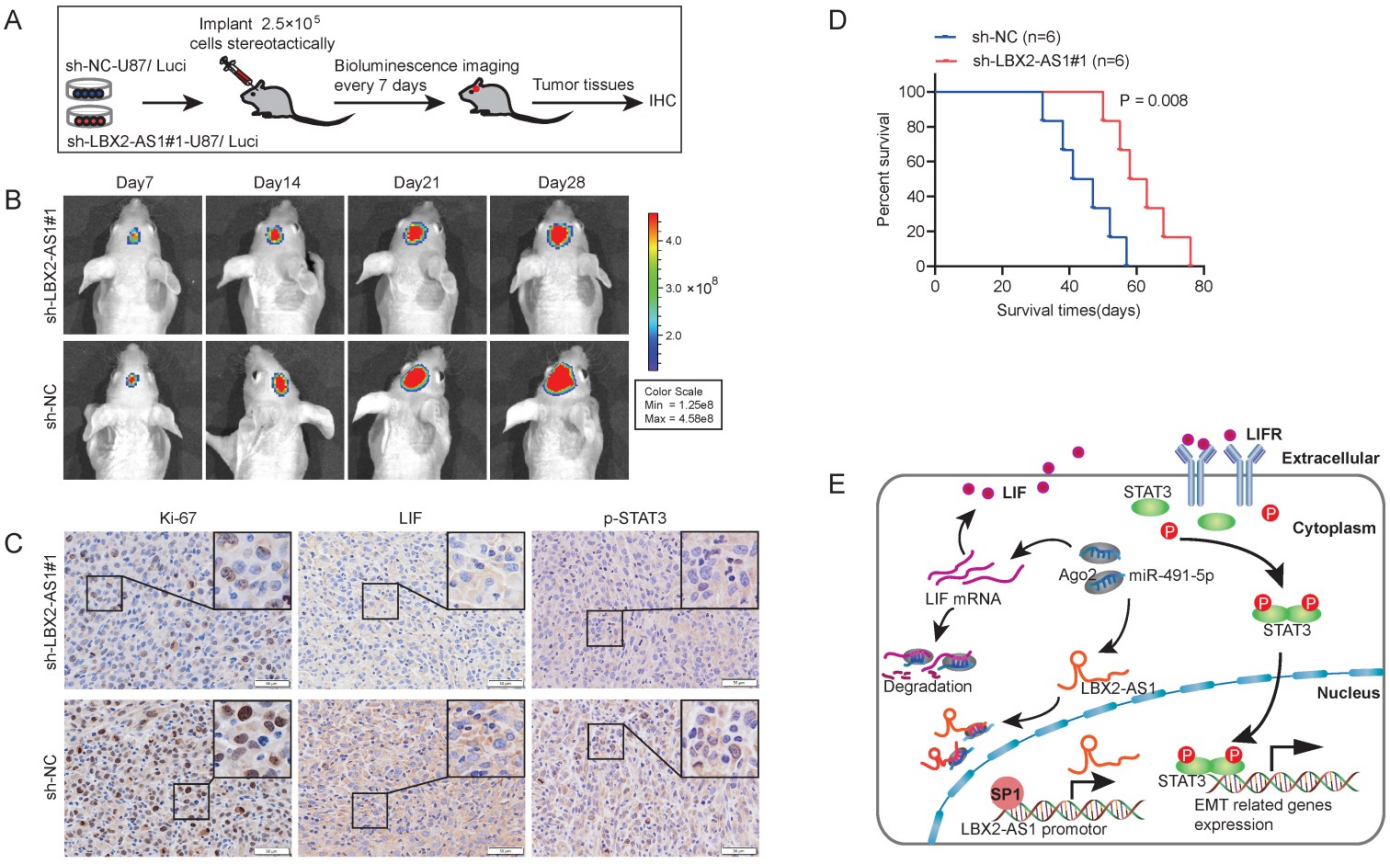
** Knockdown of LBX2-AS1 alleviates the growth of orthotopic glioma in nude mice. (A)** 2.5×10^5^ luciferase-labeled U87 cells transfected with sh-LBX2-AS1#1 or sh-NC were stereotactically implanted in nude mice. **(B)** Bioluminescence images of nude mice. **(C)** IHC staining of Ki-67, p-STAT3 and LIF in mouse brain sections of glioma. Scale bar = 50 μm. **(D)** Kaplan-Meier survival curves of orthotopic glioma mice. **(E)** Schematic diagram of mechanism of LBX2-AS1 regulating EMT in glioma.

To assess the *in vivo* function of LBX2-AS1, 2.5×10^5^ luciferase-labeled U87 cells transfected with sh-LBX2-AS1#1 or sh-NC were stereotactically implanted in nude mice, and bioluminescence images were captured every 7 days (Figure 8A). The growth of orthotopic tumor was evaluated using optical imaging. On the 14^th^, 21^st^ and 28^th^ day of implanting, the growth of orthotopic glioma was significantly alleviated by knockdown of LBX2-AS1 (Figure 8B). IHC staining revealed that positive expressions of Ki-67, LIF and p-STAT3 in tumor sections of mice with knockdown of LBX2-AS1 were significantly lower than those of controls (Figure 8C). Moreover, Kaplan-Meier survival curves revealed a prolonged survival in mice with knockdown of LBX2-AS1, confirming the oncogenic role of LBX2-AS1 in promoting the *in vivo* growth of glioma (Figure 8D).

## Discussion

Currently, a growing number of cancer associated lncRNAs have been highlighted, which are considered as potential biomarkers or therapeutic targets 23. In glioma, lncRNAs are also widely involved in the regulation of tumor progression and chemotherapy resistance 24. Through bioinformatic analyses using online databases, we found that LBX2-AS1 was upregulated in glioma, and positively correlated to tumor stage. In addition, high level of LBX2-AS1 predicted poor prognosis of patients. Notably, LBX2-AS1 level was much higher in recurrent cases and the mesenchymal (MES) subtype, indicating the involvement of LBX2-AS1 in the malignant development of glioma 25-27. The following *in vitro* experiments have demonstrated the oncogenic role of LBX2-AS1 in promoting proliferation and EMT.

Revealed by the whole transcriptome research, the expression profile of lncRNAs is more specific than that of mRNAs, which is specifically expressed in certain cell types, tissues, developmental stages or pathological states 28. It is concluded that lncRNA expression is more strictly regulated in comparison to protein-encoding genes. Considering that transcription of lncRNAs is mediated by TFs and epigenic regulatory factors, we analyzed the promoter region of LBX2-AS1 using JASPAR, and predicted binding TFs 29-31. Through dual luciferase reporter assay, SP1 was validated to highly bind to the promoter region of LBX2-AS1, and induced upregulation of LBX2-AS1. Using JASPAR and ENCODE, multiple SP1 binding sites were identified in the promoter region of LBX2-AS1. We further demonstrated that the P1 site in the promoter of LBX2-AS1 was responsible for SP1-induced transcription triggering of LBX2-AS1.

Subcellular distribution of lncRNA determines its function in regulating malignant phenotypes of cancer cells 32, 33. LBX2-AS1 was found both expressed in cell nuclei and cytoplasm, which was more pronounced in the latter. Cytoplasmic lncRNAs have been widely recognized for their ceRNA function. By competitively sponging miRNAs, lncRNAs prevent the Ago-dependent degradation of target genes that bind to miRNAs 34, 35. Here RIP assay showed that LBX2-AS1 could interact with Ago2, suggesting that LBX2-AS1 was able to exert the sponge effect on miRNAs. Subsequently, LBX2-AS1 was proven to be a potential target of miR-491-5p. It is reported that miR-491-5p can alleviate the malignant progression of glioma by inhibiting cell proliferation and invasion 36. In this study, the suppressed migratory, invasive and proliferative potentials of glioma cells by knockdown of LBX2-AS1 could be reversed by silence of miR-491-5p. Hence, we have proven that LBX2-AS1 exerted its oncogenic role in glioma by sponging miR-491-5p as a ceRNA.

According to the prediction using starBase, LIF was identified as a potential target of miR-491-5p. Previous evidences have shown the upregulation of LIF in solid tumor, which mediates the proliferation, invasion and metastasis of tumor cells 37. Serving as a cytokine in the IL-6 family, LIF can bind to the heterodimeric receptor complex on the cell membrane composed of the LIF receptor (LIFR) and the glycoprotein gp130, thereby initiating the tyrosine phosphorylation cascade of the JAK/STAT3 pathway 38, 39. Through activating the STAT3 signaling, LIF boosts the proliferative and invasive capacities of choriocarcinoma cells 40. Moreover, the self-renewal of glioma-initiating cells is achieved by the LIF/STAT3 signaling 41. Very latest, the biological significance of LIF in triggering the development and chemotherapy resistance of pancreatic ductal adenocarcinoma has been revealed 42. Previous researches have uncovered high level of LIF was correlated to proliferation and EMT in glioma, which was further supported by the findings from CCK-8, colony formation and Transwell assays in our study. Knockdown of LIF significantly reduced nuclear level of phosphorylated STAT3 in glioma cells. While, STAT3 dimerization and nuclear translocation could trigger the transcription of EMT related genes 43, 44. RIP assay confirmed that LBX2-AS1 upregulated LIF by exerting the sponge effect on miR-491-5p. Western blot results further showed that knockdown of LBX2-AS1 inhibited the activation of the LIF/STAT3 signaling, manifesting as influenced N-cadherin, E-cadherin and Vimentin, which could be abolished by knockdown of miR-491-5p. Overexpression of LBX2-AS1 yielded the opposite results, and they were, as expected, reversed by overexpressed miR-491-5p. Collectively, LBX2-AS1 and miR-491-5p regulated the expression of N-cadherin, E-cadherin and Vimentin in glioma cells by the LIF/STAT3 axis.

## Conclusion

Taken together, LBX2-AS1 is upregulated in glioma and correlated to the poor prognosis of glioma patients. It exerts the sponge effect on miR-491-5p, thus upregulating LIF and activating the LIF/STAT3 axis for promoting proliferation and EMT in glioma. In addition, SP1 is identified to activate LBX2-AS1 expression, although the underlying mechanism needs further investigation. Our findings suggested that LBX2-AS1 may be a novel diagnostic marker and therapeutic target of glioma.

## Supplementary Material

Supplementary figures and tables.
