# Supplementary material for: SP1-upregulated LBX2-AS1 promotes the progression of glioma by targeting the miR-491-5p/LIF axis
Source: J Cancer. 2021 Oct 11;12(23):6989–7002. doi: 10.7150/jca.63289 (PMC8558668; doi:10.7150/jca.63289)
Supplement: Supplementary file 1 — Supplementary figures and tables. [file jcav12p6989s1.pdf]

Supplemental materials:

Table S1: Sequences of shRNAs

	sense sequence (5'-3')	anti-sense sequence (5'-3')
**sh-LBX2-AS1#1**	GGAATGTTTGCTGAATTAA	TTAATTCAGCAAACATTCC
**sh- LBX2-AS1#2**	CGTTTGGAAGCTTTGCTAA	TTAGCAAAGCTTCCAAACG
**sh- LBX2-AS1#3**	AGGAATGTTTGCTGAATTA	TAATTCAGCAAACATTCCT

Table S2: Sequences of siRNAs

	sense sequence (5'-3')	anti-sense sequence (5'-3')
**si-SP1**	GGAUGGUUCUGGUCAAAUACA	UAUUUGACCAGAACCAUCCUG
**si-LIF#1**	GGAAGUCGACAAUGUUACAAG	UGUAACAUUGUCGACUUCCAG
**si-LIF#2**	CAGAAGAAUGGUACAAAUCCAAG	CUUGGAUUUGUACCAUUCUUCUG

Table S3: Sequences for has-miR-491-5p

	sequence (5'-3')
**hsa-miR-491-5p mimics (sense)**	AGUGGGGAACCCUUCCAUGAGG
**hsa-miR-491-5p mimics (anti-sense)**	CCUCAUGGAAGGGUUCCCCACU
**hsa-miR-491-5p inhibitor (anti-sense)**	CCUCAUGGAAGGGUUCCCCACU
**miR-NC (sense)**	UUUGUACUACACAAAAGUACUG
**miR-NC (anti-sense)**	CAGUACUUUUGUGUAGUACAAA
**Anti-NC (anti-sense)**	CAGUACUUUUGUGUAGUACAAA

Table S4: Sequences of primers used for qRT-PCR

qPCR primer name	Sequence (5'-3')
LBX2-AS1 (Forward)	CGTGGGGAATGGACCCATAG
LBX2-AS1 (Reverse)	CGAGCCTTGGTCTTGTCTGT
LIF (Forward)	CCAACGTGACGGACTTCCC
LIF (Reverse)	TACACGACTATGCGGTACAGC
CLCF1 (Forward)	TTTCAACGAGCCAGACTTCAAC
CLCF1 (Reverse)	GAGGCCACGCAAGTAACACA
SOSC3 (Forward)	CCTGCGCCTCAAGACCTTC
SOSC3 (Reverse)	GTCACTGCGCTCCAGTAGAA
miR-491-5p (Forward)	CGAGTGGGGAACCCTTCC
miR-491-5p (Reverse)	AGTGCAGGGTCCGAGGTATT
miR-6763-5p (Forward)	GCGCTGGGGAGTGGCT
miR-6763-5p (Reverse)	AGTGCAGGGTCCGAGGTATT
miR-3612 (Forward)	GCGAGGAGGCATCTTGAGA
miR-3612 (Reverse)	AGTGCAGGGTCCGAGGTATT
miR-4525 (Forward)	CGGGGGGGATGTGCATG
miR-4525 (Reverse)	AGTGCAGGGTCCGAGGTATT
U6 (Forward)	CTCGCTTCGGCAGCACA
U6 (Reverse)	AACGCTTCACGAATTTGCGT
GAPDH (Forward)	GAACGGGAAGCTCACTGG

GAPDH (Reverse)	GCCTGCTTCACCACCTTCT

Table S5: Sequences of primers used for ChIP-PCR

ChIP-PCR primer name	Sequence (5'-3')
P 1 (Forward)	GGGCCAAAGACTCCTTGACAAAG
P 1 (Reverse)	GGCCCATCAGCCTTTAAGTGTG
P 2 (Forward)	AGCCCCTTCCATCTCTCCCGCCTAG
P 2 (Reverse)	CTTAGAGGGGCCTCTGCTCTCCT
P 3 (Forward)	GTGAATGTGTAGCAGGGTTGGA
P 3 (Reverse)	GCTCCCTGAACTCCCACTGCCCT

Table S6: List of antibodies

Antibody	Catalogue NO.	Company
LIF	ab138002	Abcam (Cambridge, UK)
p-STAT3	ab76315	Abcam (Cambridge, UK)
STAT3	12640	Cell Signaling Technology (Beverly, MA)
SP1	9389	Cell Signaling Technology (Beverly, MA)
N-cadherin	13116	Cell Signaling Technology (Beverly, MA)
E-cadherin	3195	Cell Signaling Technology (Beverly, MA)
Vimentin	5741	Cell Signaling Technology (Beverly, MA)
GAPDH	5174	Cell Signaling Technology (Beverly, MA)

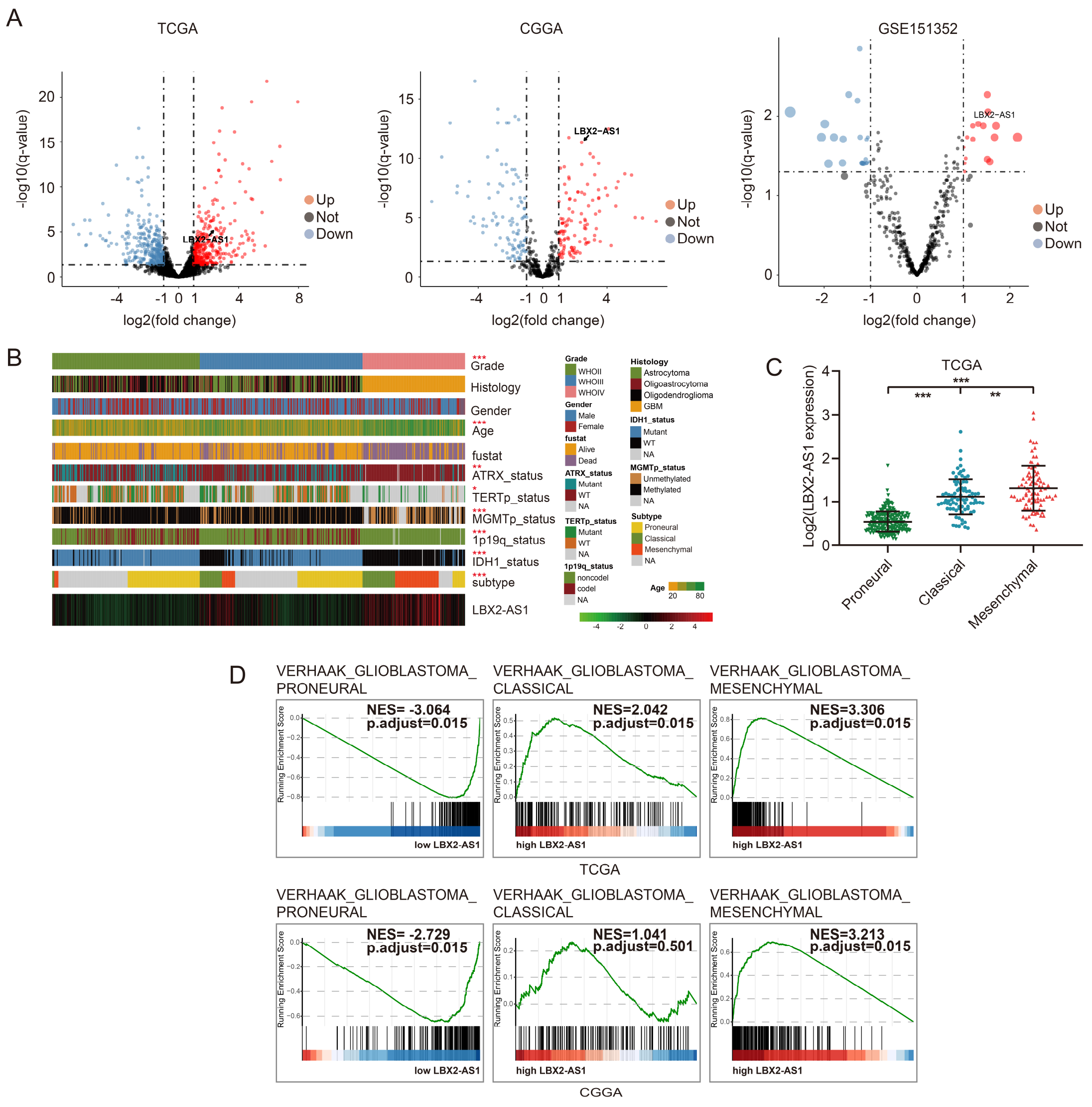

Figure S1 LBX2-AS1 is upregulated in glioma. (A) Volcano plots of differentially expressed lncRNAs between GBM and normal samples from TCGA, CGGA and GSE151352 databases. (B) Heatmap of the associations between LBX2-AS1 and clinicopathological features of glioma in TCGA dataset. (C) Relative levels of LBX2-AS1 in glioma samples from TCGA dataset categorized by transcription subtypes. (D) GSEA revealed the correlation between LBX2-AS1 and the transcription subtypes in glioma. **p*<0.05, ***p*<0.01, ****p*<0.001.

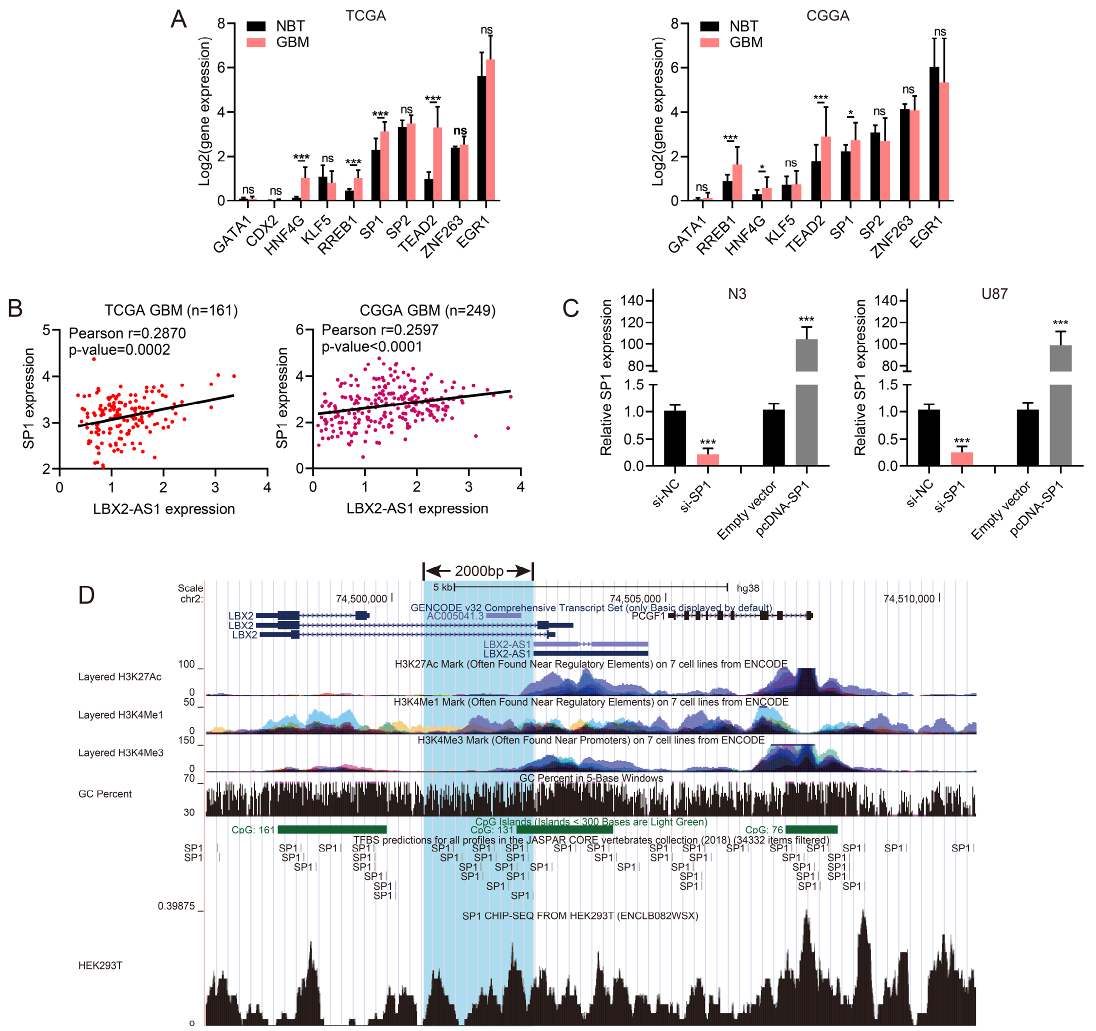

Figure S2 SP1 upregulated LBX2-AS1 in glioma. (A) Relative levels of the top 10 scored transcription factors that could bind to the promoter region of LBX2-AS1 in TCGA and CGGA databases. (B) Correlation between the expression level of SP1 and LBX2-AS1 in TCGA and CGGA databases. (C) Transfection efficacy of si-SP1 and pcDNA-SP1 in N3 and U87 cells. (D) Highly enriched SP1 in the promoter region of LBX2-AS1 (highlighted in blue) in JASPAR and ENCODE. *p<0.05, ***p<0.001.

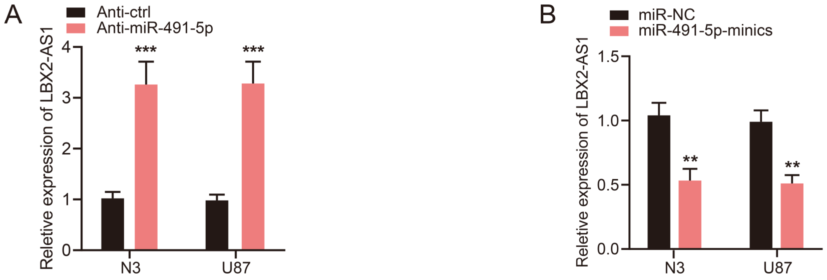

Figure S3 The expression level of LBX2-AS1 in miR-491-5p and anti-miR-491-5p overexpressed cells detected by qPCR. (A) qPCR analysis detected LBX2-AS1 expression in N3 and U87 cells transfected with anti-miR-491-5p. (B) qPCR analysis probed LBX2-AS1 expression in N3 and U87 cells transfected with miR-491-5p mimics. **p<0.01, ***p<0.001.

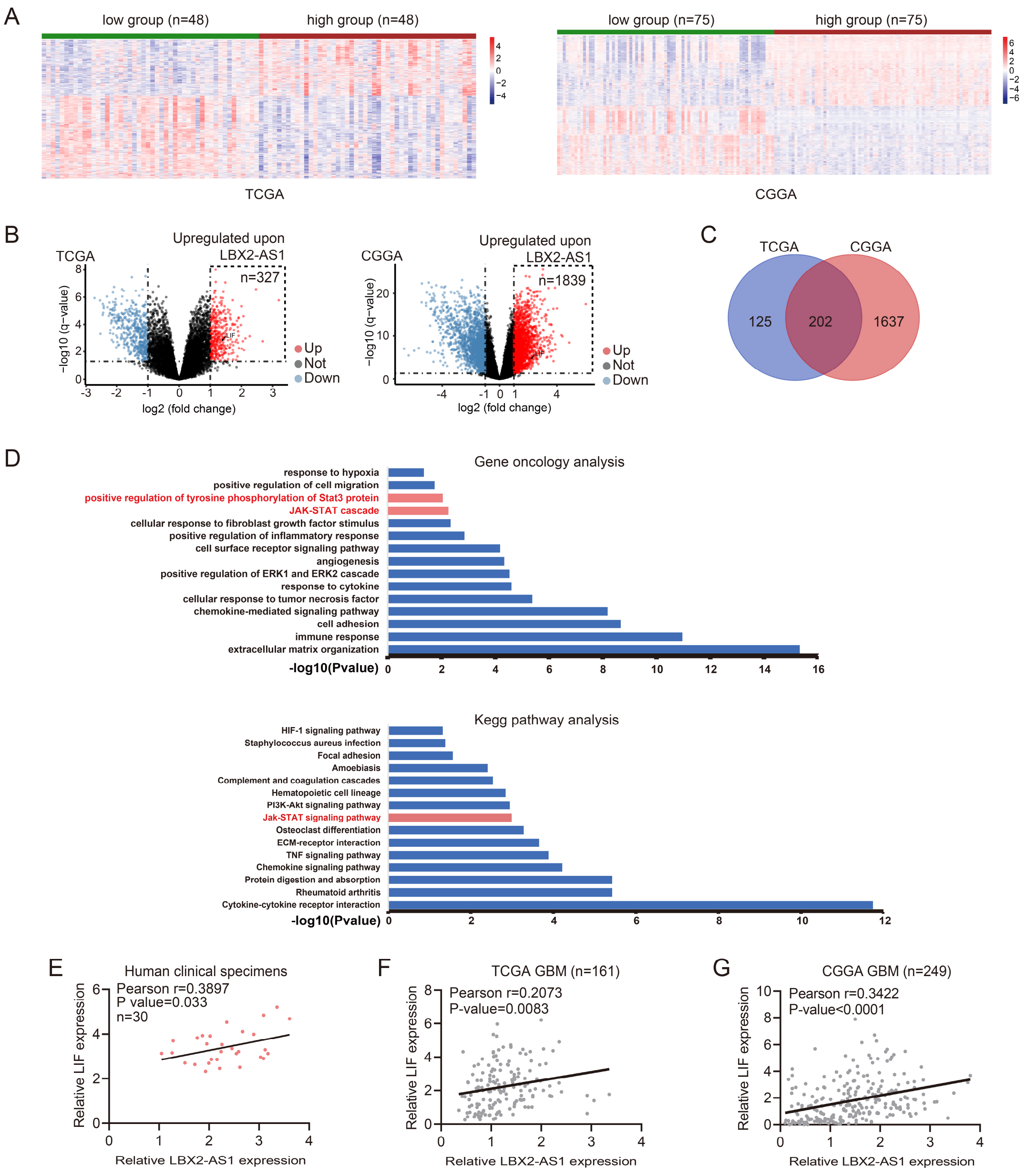

Figure S4 LIF is the target gene in the ceRNA network of LBX2-AS1/ miR-491-5p. (A) Heatmaps of differentially expressed mRNAs in GBM samples from TCGA and CGGA databases categorized into high group and low group based on expression level of LBX2-AS1. (B) Volcano plots showing genes upregulated by LBX2-AS1 in TCGA and CGGA. (C) The intersection of upregulated genes regulated by LBX2-AS1. (D) GO and KEGG showed LBX2-AS1 was mainly enriched in the JAK-STAT3 signaling pathway. (E) Correlation between the expression level of LIF and LBX2-AS1 in 30 clinical specimens of GBM. (F, G) Correlation between the expression level of LIF and LBX2-AS1 in TCGA and CGGA databases.
